# Protection of melatonin against acidosis‐induced neuronal injuries

**DOI:** 10.1111/jcmm.15351

**Published:** 2020-05-04

**Authors:** Yan Shi, Er‐Li Cai, Can Yang, Chao‐Yuan Ye, Peng Zeng, Xiao‐Ming Wang, Ying‐Yan Fang, Zhi‐Kang Cheng, Qun Wang, Fu‐Yuan Cao, Xin‐Wen Zhou, Qing Tian

**Affiliations:** ^1^ Department of Pathology and Pathophysiology School of Basic Medicine Tongji Medical College Huazhong University of Science and Technology Wuhan China; ^2^ Key Laboratory of Neurological Disease of National Education Ministry Institute for Brain Research Huazhong University of Science and Technology Wuhan China; ^3^ School of Medicine Hunan Normal University Changsha China; ^4^ Department of Emergency Surgery Tongji Medical College Union Hospital Huazhong University of Science and Technology Wuhan China

**Keywords:** acidosis, dendritic damage, melatonin, neuron

## Abstract

Acidosis, a common feature of cerebral ischaemia and hypoxia, plays a key role in these pathological processes by aggravating the ischaemic and hypoxic injuries. To explore the mechanisms, in this research, we cultured primary neurons in an acidic environment (potential of hydrogen [pH]6.2, 24 hours) to mimic the acidosis. By proteomic analysis, 69 differentially expressed proteins in the acidic neurons were found, mainly related to stress and cell death, synaptic plasticity and gene transcription. And, the acidotic neurons developed obvious alterations including increased neuronal death, reduced dendritic length and complexity, reduced synaptic proteins, tau hyperphosphorylation, endoplasmic reticulum (ER) stress activation, abnormal lysosome‐related signals, imbalanced oxidative stress/anti‐oxidative stress and decreased Golgi matrix proteins. Then, melatonin (1 × 10^−4^ mol/L) was used to pre‐treat the cultured primary neurons before acidic treatment (pH6.2). The results showed that melatonin partially reversed the acidosis‐induced neuronal death, abnormal dendritic complexity, reductions of synaptic proteins, tau hyperphosphorylation and imbalance of kinase/phosphatase. In addition, acidosis related the activations of glycogen synthase kinase‐3β and nuclear factor‐κB signals, ER stress and Golgi stress, and the abnormal autophagy‐lysosome signals were completely reversed by melatonin. These data indicate that melatonin is beneficial for neurons against acidosis‐induced injuries.

## 1 INTRODUCTION

The brain is a highly energy‐consuming organ, expending more energy than any other organ in proportion to its size. When the energy delivery, production, utilization and storage of brain are insufficient or impaired, the acid‐base disturbance, especially acidosis, will occur. Clinically, brain acidosis often results either from an increase in tissue partial pressure of carbon dioxide (PCO_2_) during hypercapnia, or from the accumulation of the by‐products of anaerobic metabolism, such as lactate and protons, during hypoxia.^1^ The extracellular potential of hydrogen (pH) will drop to 6.5‐6.0 during ischaemia in normoglycaemic conditions and even below 6.0 in severe ischaemia or hyperglycaemic conditions.^1^, ^2^ In brain, the acid‐base equilibrium is essential for neurotransmission and neuronal excitability. Gamma‐aminobutyric acid A (GABAA) receptors, various voltage‐gated ion channels and transient receptor potential vanilloid 1 are modulated by an acidic pH.^3^ Almost all in vivo studies have shown that acidosis is a common feature of ischaemia and further aggravates the damage, resulting in disturbance in ion homeostasis, excitotoxicity and a number of cell death‐mediating pathways, which are responsive to inflammatory and oxidative stress mediators ultimately neuronal death.^4^, ^5^ It was previously shown that lactic acidosis in astrocytes under ischaemic conditions accelerated hypoxia and neuronal damage, leading to more severe infarctions.^6^ A reduced pH has also been implicated in seizures,^7^ Down's syndrome,^8^ Pick's disease^8^ and neurodegenerative diseases including Alzheimer's disease (AD) and Huntington's disease.^8^ Thus, to elucidate the acidosis‐induced neuronal injuries is critical for brain protection from acidosis.

Melatonin (N‐acetyl‐5‐methoxytryptamine), a tryptophan metabolite synthesized mainly in the pineal gland produced primarily by the pineal gland at night, has multiple biological activities, including antioxidants, anti‐inflammatory, neuroprotection, anti‐depression, antinociception and antianxiety.^9^ In addition, it is also important in stabilizing the structures of synapses and enhancing the functions of synapses, and further benefit to cognition.^10^ Although the mechanism is still unclear, studies have shown that melatonin has protective effects on the hypercapnia and hypoxia sensitivity in rats.^11^, ^12^ It has been found that acute foetal hypoxia induced cerebral oxidative stress and inflammation in the late gestation brain, while melatonin abolished the primary and secondary increases in brain hydroxyl radical formation and reduced cerebral lipid peroxidation, thereby reducing brain damage.^13^, ^14^ In addition, systemic or transdermal neonatal melatonin administration significantly reduced the neuropathology and encephalopathy signs associated with perinatal asphyxia.^15^ Other studies have shown that melatonin reduced oxidative stress and cell damage in the foetal sheep brain in response to severe hypoxia^16^ and protected against hippocampal cell loss following cerebral ischaemia and reperfusion in foetal and neonatal rats by improving mitochondrial injury.^17^ In this study, we studied the effect of melatonin pre‐treatment on the acidosis‐induced neuronal injuries in vitro.

The primary neurons were cultured in an acidic environment (pH6.2) for 24 hours to mimic the acidosis after growing in the normal medium for 14 days. By proteomic analysis, 69 differentially expressed proteins were found, which were mainly related to stress and cell death, synaptic plasticity and gene transcription. Then, before being cultured in the acidic medium, the primary neurons were exposed in melatonin (1 × 10^−4^ mol/L, Mel neurons) or dimethyl sulphoxide (DMSO) (0.01%, Veh neurons) for 24 hours. It was observed that melatonin pre‐treatment increased the neuronal cell viability, and the total length and the complexity of dendrites, and reversed the abnormalities of synaptic proteins and tau hyperphosphorylation caused by acidosis. Compared with the neurons cultured in the normal medium (Con neurons), the increased levels of nuclear factor‐κB (NF‐κB) p65, glucose‐regulated protein 78 (GRP78/Bip), activating transcription factor6 (ATF6) and the inhibition of nuclear factor erythroid 2–related factor 2 (Nrf2) were found in Veh neurons, but not in Mel neurons. Thus, all these in vitro data of this study illustrated that melatonin is beneficial for neurons against acidosis‐induced injuries.

## 2 MATERIALS AND METHODS

### 2.1 Drugs and antibodies

Melatonin (N‐acetyl‐5‐methoxytryptamine) was procured from Sigma (St. Louis, MO, USA) and dissolved in DMSO (0.1 mol/L). The primary antibodies used for immunofluorescence staining and Western blotting are listed in Table 1.

**TABLE 1:** Antibodies employed in this study and their properties

Antibody	Epitopes	mAb/pAb	Dilution	Source
MAP2	Total MAP2	pAb	1:1000 (IF)	Abcam (Cambridge, UK)
NF‐κB p65	p‐NF‐κB p65 at Ser536	pAb	1:1000 (WB)	Cell Signaling Technology (Danvers, MA, USA)
p‐Nrf2	p‐Nrf2 at Ser40	pAb	1:1000 (WB)	Abcam
DM1A	α‐tubulin	mAb	1:2000 (WB)	Abcam
NR2A	Total NR2A	pAb	1:1000 (WB)	Cell Signaling Technology
NR2B	Total NR2B	pAb	1:1000 (WB)	Cell Signaling Technology
NR1	Total NR1	pAb	1:1000 (WB)	Millipore (Billerica, MA, USA)
GluR1	Total GluR1	pAb	1:1000 (WB)	Millipore
GluR2	Total GluR2	mAb	1:1000 (WB)	Millipore
Synapsin 1	Total Total synapsin 1	pAb	1:1000 (WB)	Millipore
Syntaxin1	Total syntaxin 1a	pAb	1:1000 (WB)	Abcam
Synaptophysin	Total synaptophysin	pAb	1:1000 (WB)	Abcam
SNAP25	Total SNAP25	mAb	1:1000 (WB)	Cell Signaling Technology
VAMP3	Total VAMP3	pAb	1:1000 (WB)	Thermo Fisher Scientific
PSD95	Total PSD95	pAb	1:500 (WB)	Cell Signaling Technology
Tau‐5	Total tau	mAb	1:1000 (WB)	Abcam
pT231	p‐tau at Thr231	pAb	1:1000 (WB)	Signalway Antibody
pS396	p‐tau at Ser396	mAb	1:1000 (WB)	Cell Signaling Technology
ps214	p‐tau at Ser214	pAb	1:1000 (WB)	Signalway Antibody
pS404	p‐tau at Ser404	pAb	1:1000 (WB)	Signalway Antibody
pT205	p‐tau at Thr205	pAb	1:1000 (WB)	Cell Signaling Technology
Tau‐1	dep‐tau at Ser198/199/202	mAb	1:1000 (WB)	Millipore
t‐GSK3β	Total GSK‐3β	pAb	1:1000 (WB)	Cell Signaling Technology
pGSK3β	p‐GSK‐3β at Ser9	pAb	1:1000 (WB)	Cell Signaling Technology
t‐AKT	Total AKT	pAb	1:1000 (WB)	Cell Signaling Technology
p‐AKT	p‐AKT at Ser473	pAb	1:1000 (WB)	Cell Signaling Technology
ERK	Total MAPK ERK1/2	pAb	1:1000 (WB)	Cell Signaling Technology
p‐ERK	p‐ERK at Thr202/Tyr204	pAb	1:1000 (WB)	Cell Signaling Technology
PP2Ac	PP2A catalytic subunit	pAb	1:1000 (WB)	Biosource (Camarillo, CA, USA)
p‐PP2Ac	p‐PP2A at Tyr307	pAb	1:1000 (WB)	Santa Cruz (Dallas, TX, USA)
Beclin 1	Total Beclin 1	pAb	1:1000 (WB)	Abcam
LC3	Total LC3	mAb	1:1000 (WB)	Cell Signaling Technology
Rab7	Total Rab7	mAb	1:1000 (WB)	Cell Signaling Technology
Lamp1	Total Total Lamp1	mAb	1:1000 (WB)	Abcam
Lamp2	Total Lamp2	mAb	1:1000 (WB)	Abcam
GRP78	Total GRP78	pAb	1:1000 (WB)	Abcam
ATF6	Total ATF6	pAb	1:1000 (WB)	Abcam
TGN46	Total TGN46	mAb	1:1000 (WB)	Abcam
GRASP55	Total GRASP55	mAb	1:1000 (WB)	Santa Cruz
GRASP65	Total GRASP65	mAb	1:1000 (WB)	Abcam
Golgin 84	Total golgin 84	mAb	1:1000 (WB)	Santa Cruz
Cas3	Total caspase‐3	mAb	1:1000 (WB)	Cell Signaling Technology
C‐cas3	Total cleaved caspase‐3	mAb	1:1000 (WB)	Cell Signaling Technology

Abbreviations: IF, immunofluorescence; mAb‐, monoclonal antibody; p‐, phosphorylated; pAb‐, polyclonal antibody; WB, Western blotting.

### 2.2 Primary neuron culture

Timed‐pregnant Sprague‐Dawley (SD) rats (No. 42009800001739; Research Resource Identifier: RGD_70508) weighing 250‐300 g each were supplied by the Experimental Animal Center of Tongji Medical College, Huazhong University of Science and Technology. All efforts were made to minimize animal suffering and to reduce the number of rats used. All experimental procedures in this research have been approved by the Animal Care and Use Committee of Huazhong University of Science and Technology.

Primary cortical neurons were prepared from 17‐ or 18‐day‐old rat embryos. The cortex of embryos were dissected and gently minced in Hank's buffered saline solution supplemented with 2% (vol/vol) glucose, trypsinized at 37°C for 15 minutes and filtered through 75‐μm nylon tamis. Then, DMEM/F12 medium containing 10% (vol/vol) foetal calf serum was added to the tissue and centrifuged at 1000 × g for 5 minutes. Dissociated neurons were plated onto poly‐D‐lysine‐coated (Sigma) coverslips in a 12‐well plate or tissue culture dish covered with a glass bottom at a higher density (400‐600 neurons/mm^2^) for Western blotting and a lower density (100‐200 neurons/mm^2^) for immunofluorescence. The cells were maintained at 37°C in a humidified incubator with 5% CO_2_. Two hours later, the medium was changed to a maintenance medium of Neurobasal containing 2% (vol/vol) B27 and 1% (vol/vol) GlutaMAX, and half of the medium was replaced with GlutaMAX‐free maintenance medium every 3 days. The primary neurons were then cultured in normal medium (pH = 7.5) for 14 days in vitro (14 DIV). The desired media with different pH values (pH6.5, pH6.2 and pH6.0) were prepared by titrating with HCl/NaOH to Neurobasal medium. All cell culture reagents were purchased from Thermo Fisher Scientific (Waltham, MA, USA).

### 2.3 Cell counting kit‐8 assay

The cell suspension was added to a 96‐well plate at a suitable density and maintained at 37°C in a humidified incubator with 5% CO_2_. The cells were then exposed to different experimental conditions. Finally, the medium was changed to fresh medium containing 10 µL/well of Cell counting kit‐8 (CCK8) solution (Dojindo Laboratories, Kumamoto, Japan), the cells were incubated in the dark for 30 minutes, and the optical densities were measured by a BioTek Synergy 2 microplate reader (Winooski, VT, USA).

### 2.4 TdT‐mediated dUTP nick end labelling staining

The cultured neurons were fixed in 4% (wt/vol) paraformaldehyde (PFA) for 15 minutes and washed with phosphate‐buffered saline (PBS) containing 0.1% (wt/vol) Triton X‐100 for 15 minutes. After incubating for 2 minutes in 0.1% Na‐Citrate supplemented with 0.1% (wt/vol) Triton X‐100, the cells were washed in PBS for 15 minutes. Then, 50 μL of TdT‐mediated dUTP nick end labelling (TUNEL) reagent (Invitrogen, Carlsbad, CA, USA) was added to each slide and incubated at 37°C for 30 minutes, followed by washing with PBS for 15 minutes. Finally, 4′,6‐diamidino‐2‐phenylindole (DAPI) was added for 10 minutes and washed with PBS for 15 minutes. Images were captured using a fluorescence microscope (Olympus, Tokyo, Japan).

### 2.5 Enzyme‐linked immunosorbent assay (ELISA)

The neuronal superoxide dismutase 1 (SOD1) levels were assayed by ELISA. The neurons were washed with PBS for 5 minutes, the neurons were digested by trypsin, and the cells were lysed and centrifuged at 5000 × g for 10 minutes at 4°C to obtain the supernatant. The neuronal supernatants were incubated in the ELISA microplate (Elabscience Biotechnology Co., Ltd, Wuhan, China) for 90 minutes at 37°C. After adding the stop solution, the optical densities were measured by a BioTek Synergy 2 microplate reader.

### 2.6 Proteomic analysis

The integrated approach used to quantify dynamic changes in the primary cultured rat neurons included isobaric tags for relative and absolute quantification (iTRAQ), high‐performance liquid chromatography (HPLC) fractionation and mass spectrometry–based quantitative proteomics. The cellular proteins were digested by trypsin, and the obtained peptides from the denatured protein were labelled by iTRAQ 6‐plex reagents. After labelled, the peptides were fractionated by high‐pH reversed‐phase HPLC. For high‐performance liquid chromatography/tandem mass spectrometry (LC‐MS/MS) analysis, the peptides were dissolved in 0.1% (vol/vol) formic acid, loaded onto a reversed‐phase pre‐column (Acclaim PepMap 100; Thermo Fisher Scientific) and then separated using a reversed‐phase analytical column (Acclaim PepMap RSLC; Thermo Fisher Scientific). The resulting peptides were analysed through a Q Exactive^™^ Plus hybrid quadrupole‐Orbitrap mass spectrometer (Thermo Fisher Scientific). To identify and quantify proteins, the resulting MS/MS data were analysed by MaxQuant with an integrated Andromeda search engine, and tandem mass spectra were searched against the UniProt rat protein database. As reported, the proteins with a fold change of >1.20 or <0.83 (with *P* < 0.05) were considered to be significantly differentially expressed.^18^

### 2.7 Western blotting

Protein concentrations were measured using a bicinchoninic acid kit (Thermo Fisher Scientific) after extracted protein from neuron. Samples were boiled at 100°C for 2 minutes and added loading buffer containing β‐mercaptoethanol and bromophenol blue. The proteins were electrophoresed by 10% sodium dodecyl sulphate‐polyacrylamide gel electrophoresis and transferred onto nitrocellulose membranes (Amersham Biosciences, Pittsburgh, PA, USA), and then, the membranes were blocked with 5% non‐fat milk dissolved in Tris‐buffered saline (TBS) containing 50 mmol/L Tris‐HCl, pH7.6, and 150 mmol/L NaCl for 1 hour and incubated with primary antibody (Table 1) at 4°C overnight. Membranes were then incubated with anti‐rabbit or antimouse IgG conjugated to IRDye^®^ 800 CW (1:10 000; Li‐Cor Bioscience, Lincoln, NE, USA) for 1 hour at room temperature away from light and washed with TBS‐Tween‐20 for 15 minutes. After scrubbed with TBS‐Tween‐20, the membranes were visualized and quantitatively analysed by the Odyssey Infrared Imaging System (Li‐Cor Bioscience).

### 2.8 Immunofluorescence staining

The cultured neurons were washed in PBS for 5 minutes, fixed in 4% PFA for 30 minutes and then washed with PBS containing 0.1% Triton X‐100 for 15 minutes. After being ruptured membrane in PBS supplemented with 0.5% Triton X‐100 for 10 minutes, the cells were incubated in 3% BSA to block non‐specific sites for 30 minutes at 25°C and then washed with PBS for 30 minutes. The cells were incubated with the primary antibodies at 4°C for 24 hours. The cells were then washed with PBS for 30 minutes and were subsequently incubated with secondary antibodies for 1 hour at 37°C. The cells were incubated with DAPI for 10 minutes and sealed with 50% glycerine after being washed with PBS. The images were observed by a laser confocal microscope (LSM780; Zeiss, Heidelberg, Germany), and the fluorescence images were analysed by the software affiliated.

### 2.9 Simple neurite tracer analysis and Sholl analysis

For assessment of neuron morphology and neuronal complexity, neurons were immunofluorescence staining with microtubule‐associated protein 2 (MAP2) according to the protocol. A z stack of the optical section was captured using Carl Zeiss LSM780 confocal microscope. The dendritic morphology of individual neurons was quantified by an individual blinded to experimental conditions using ImageJ software (NIH, Bethesda, MA, USA) with the Sholl analysis plug‐in as previously described.^19^ Using the centre of the soma as reference point, dendritic length and branch points were measured as a function of radial distance from the soma by adding up all values in each successive concentric segment. Total dendritic length and number of branch points were analysed for each neuron.

### 2.10 Statistical analysis

Data were presented as means ± SEM. Statistical analysis was performed using SPSS 12.0 (SPSS Inc, Chicago, IL, USA). Statistical analysis was performed by using the unpaired Student's *t* test or one‐way ANOVA followed by the Tukey post hoc test. Null hypotheses were rejected at *P* < 0.05.

## 3 RESULTS

### 3.1 Decreases in extracellular pH value induced neuronal injury

**FIGURE 1:**
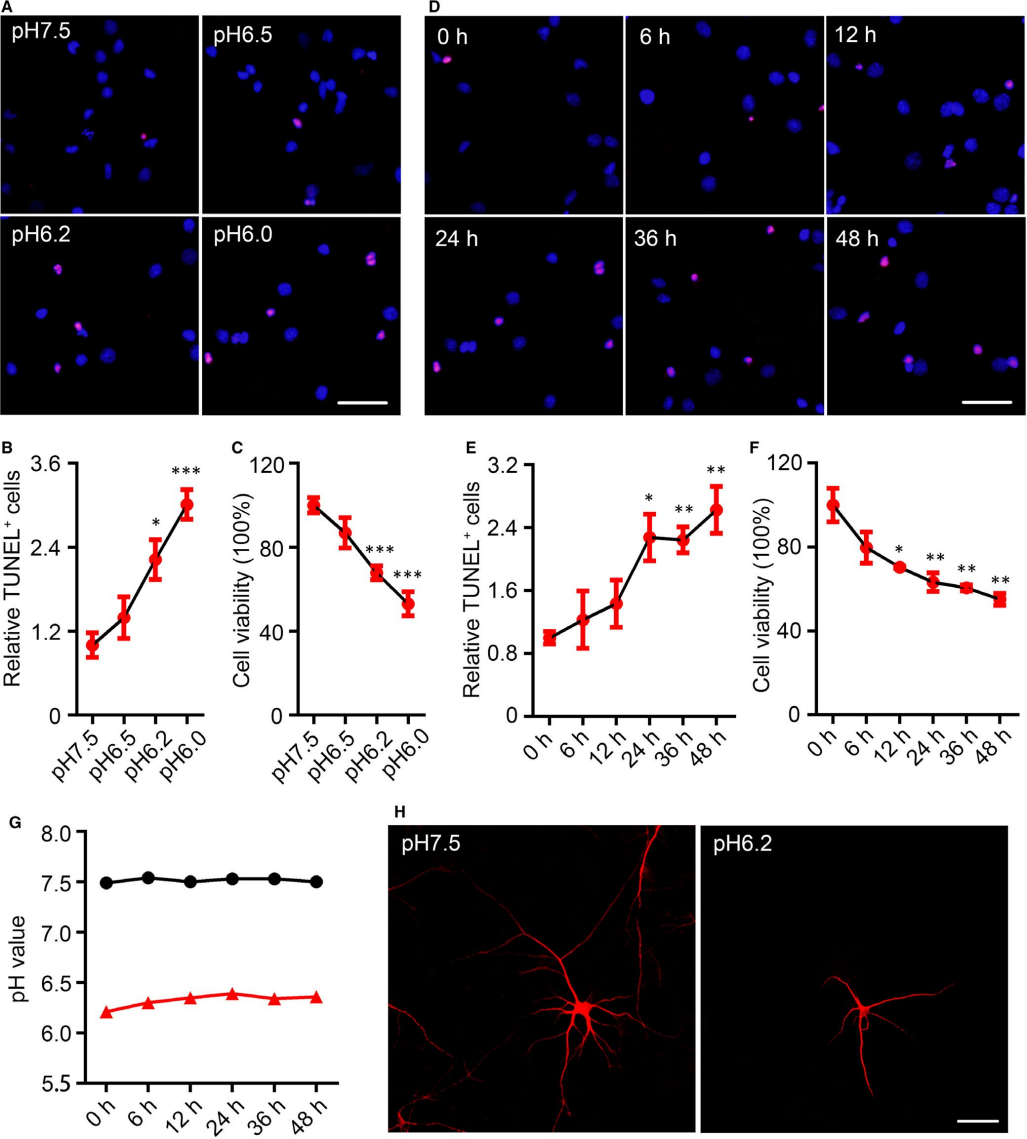
Decreases in extracellular pH value induced neuronal injury. A, TdT‐mediated dUTP nick end labelling (TUNEL) staining (red) in cultured rat cortical neurons (14‐days in vitro, 14 DIV) was performed after acid treatments (pH6.5, pH6.2 and pH6.0) for 24 h (scale bar = 50 μm) and quantified (B). Data were presented as means ± SEM. *P < 0.05, ***P < 0.001 vs Con, n = 4/group. C, The relative cell viabilities of rat cortical neurons after acidic treatment for 24 h were shown by Cell Counting Kit‐8 (CCK8) assay. Data were presented as means ± SEM. ***P < 0.001 vs Con, n = 6/group. D, TUNEL staining in the rat cortical neurons (14 DIV) was performed after pH6.2 treatment for 0‐48 h (eg 0, 6, 12, 24, 36 and 48 h, respectively) (scale bar = 50 μm) and quantified (E). Data were presented as means ± SEM. *P < 0.05, **P < 0.01 vs Con, n = 4/group. F, The relative cell viabilities of rat cortical neurons after pH6.2 treatment for 0‐48 h were shown by CCK8 assay. Data were presented as means ± SEM. *P < 0.05, **P < 0.01 vs Con, n = 6/group. G, Changes in acidity of normal medium and pH6.2 medium within 48 h. H, Images for observing neurons were collected on a confocal laser scanning microscope after immunofluorescence staining with an antibody recognizing microtubule‐associated protein 2. Scale bar = 50 μm

After growing in the normal medium (pH = 7.5) for 14 DIV, the primary neurons were cultured in the medium for 24 hours with different pH values, for example pH6.5, pH6.2 and pH6.0. As the numbers of TUNEL‐positive neurons were significantly increased (Figure 1A,B) and the relative cell viabilities were significantly decreased (Figure 1C) in the medium of pH6.2 and pH6.0, we chose the medium of pH6.2 for long‐term culture. At 36 and 48 hours after growing in the medium of pH6.2, the numbers of TUNEL‐positive neurons were increased too, but showed no difference when compared with culturing 24 hours (Figure 1D,E). The decreased cell viabilities were observed from 12 to 48 hours (Figure 1F). Meanwhile, we monitored the pH values of the medium at different time points and ensured the stabilization of the culture (Figure 1G). By immunofluorescence staining of MAP2, the neurons had less neurite numbers and decreased neurite length when cultured in medium of pH6.2 for 24 hours (Figure 1H).

**FIGURE 2:**
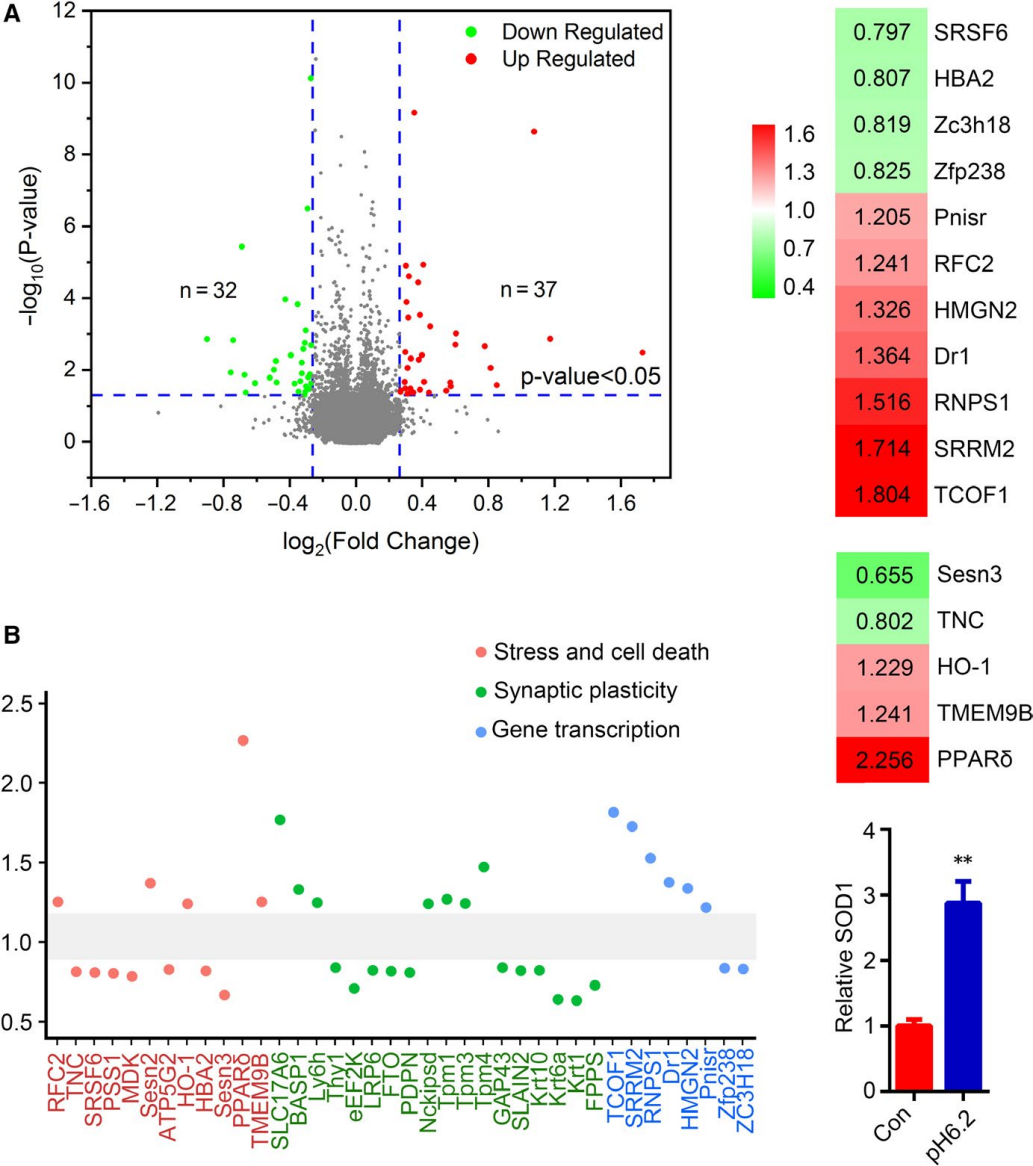
Classification of differentially expressed proteins of pH6.2 neurons. A, A total of 69 differentially expressed proteins were obtained in this study. Among them, there are 37 proteins were up‐regulated (ratio > 1.2, *P *< 0.05), and 32 proteins were down‐regulated (ratio < 0.83, *P* < 0.05) in pH6.2/Con. B, By biological functions, the differentially expressed proteins were classified into three groups, for example stress and cell death, synaptic plasticity and gene transcription. C, The differentially expressed proteins related to gene transcription are listed with a ratio (pH6.2/Con), including replication factor C subunit 2 (RFC2), serine/arginine‐rich splicing factor 6 (SRSF6), haemoglobin subunit α 2 (HBA2), treacle ribosome biogenesis factor 1 (TCOF1), serine/arginine repetitive matrix 2 (SRRM2), RNA‐binding protein with serine‐rich domain 1 (RNPS1), down‐regulator of transcription 1 (Dr1), non‐histone chromosomal protein HMG‐17 (HMGN2), Pnisr, zinc finger and BTB domain‐containing protein 18 (Zfp238) and zinc finger CCCH domain‐containing protein 18 (ZC3H18). D, The differentially expressed proteins related to oxidative stress are listed with a ratio (pH6.2/Con), including tenascin C (TNC), haeme oxygenase 1 (HO‐1), sestrin 3 (Sesn3), transmembrane protein 9B (TMEM9B) and peroxisome proliferator–activated receptor delta (PPARδ). Red colour indicates the increased proteins (*P *< 0.05), and green indicates the decreased ones (*P *< 0.05). E, Levels of superoxide dismutase 1 (SOD1) in neurons were detected by ELISA. Data were presented as means ± SEM (n = 4/group). **P < 0.01 vs Con

We quantitatively analysed the differentially expressed proteins in the neurons cultured in medium of pH6.2 for 24 hours compared with those in normal medium. In total, 6792 protein groups were identified, among which 5324 proteins were quantified. Using an iTRAQ ratio of >1.2 coupled with *P* < 0.05 as the up‐regulated threshold and <0.83 coupled with *P* < 0.05 as the down‐regulated threshold, 69 differentially expressed proteins were obtained, including 37 up‐regulated and 32 proteins down‐regulated proteins (Figure 2A). By biological functions assay, we found the functions of lots of differentially expressed proteins mainly focused on three aspects, for example stress and cell death, synaptic plasticity and gene transcription (Figure 2B). In pH6.2 neurons, replication factor C subunit 2 (RFC2) was increased (1.241, *P* = 0.0088), and the levels of serine/arginine‐rich splicing factor 6 (SRSF6) (0.797, *P* = 0.0062) and haemoglobin subunit α 2 (HBA2) (0.807, *P* = 0.0018) were decreased. RFC2, SRSF6 and HBA2 are included in the stress and cell death related proteins. The levels of treacle ribosome biogenesis factor 1 (TCOF1) (1.804, *P* = 0.027), serine/arginine repetitive matrix 2 (SRRM2) (1.714, *P* = 0.0022), RNA‐binding protein with serine‐rich domain 1 (RNPS1) (1.516, *P* = 0.0020), down‐regulator of transcription 1 (Dr1) (1.364, *P* = 0.00062), non‐histone chromosomal protein HMG‐17 (HMGN2) (1.326, *P* = 0.00062) and Pnisr (1.205, *P* = 0.040) were increased, and the levels of zinc finger and BTB domain‐containing protein 18 (Zfp238) (0.825, *P* = 0.013) and zinc finger CCCH domain‐containing protein 18 (ZC3H18) (0.819, *P* = 0.016) were decreased in pH6.2 neurons. These proteins are gene transcription–related (Figure 2C).

### 3.2 Melatonin impeded neuronal apoptosis and dendritic abnormalities in acidic condition

Several oxidative stress–related molecules were included in the differentially expressed proteins (Figure 2D). Tenascin C (TNC), a glycoprotein expressed in the extracellular matrix of various tissues during development, disease or injury and in restricted neurogenic areas of brain, was decreased in the pH6.2 neurons (0.802, *P* = 0.0026). Deficiency of TNC alleviates neuronal apoptosis and neuroinflammation in mice.^20^ Haeme oxygenase 1 (HO‐1), a marker of oxidative stress and responsive to many stimuli such as reactive oxygen species (ROS), modified lipids and inflammatory cytokines,^21^, ^22^ was increased in the pH6.2 neurons (1.229, *P* = 0.0032). Sestrin 3 (Sesn3), a molecular responsible for the ROS accumulation and also an autophagy mediator,^23^, ^24^ was decreased in the pH6.2 neurons (0.655, *P* = 0.023). It was reported knockdown of Sesn3 caused an increase of FOXO3‐induced ROS and accelerated apoptosis.^23^ Transmembrane protein 9B (TMEM9B), a glycosylated protein mainly localized in membranes of the lysosome and an upstream activator of the NF‐κB signalling,^25^, ^26^ was increased in the pH6.2 neurons (1.241, *P* = 0.046). The level of peroxisome proliferator–activated receptor delta (PPARδ) was increased in the pH6.2 neurons (2.256, *P* = 0.0013), activation of which was reported prevent oxidative stress and inflammation in several neurodegeneration models.^27^, ^28^ Sesn2, an antioxidant protein activated under stress to protect neurons by decreasing the expression of the Nrf2,^29^ was increased in the pH6.2 neurons (1.358, *P* = 0.042). SOD1, an antioxidant enzyme, is an essential antioxidant located in the cytosol and inter‐membrane space of the mitochondria that functions to form hydrogen peroxide from the dismutation of the superoxide radical.^30^ By ELISA, the SOD1 level was found increased in the pH6.2 neurons (Figure 2E). All these data indicated the oxidative stress in the pH6.2 neurons. Thus, melatonin, a well‐defined antioxidant, was used to pre‐treat the cultured neurons.

**FIGURE 3:**
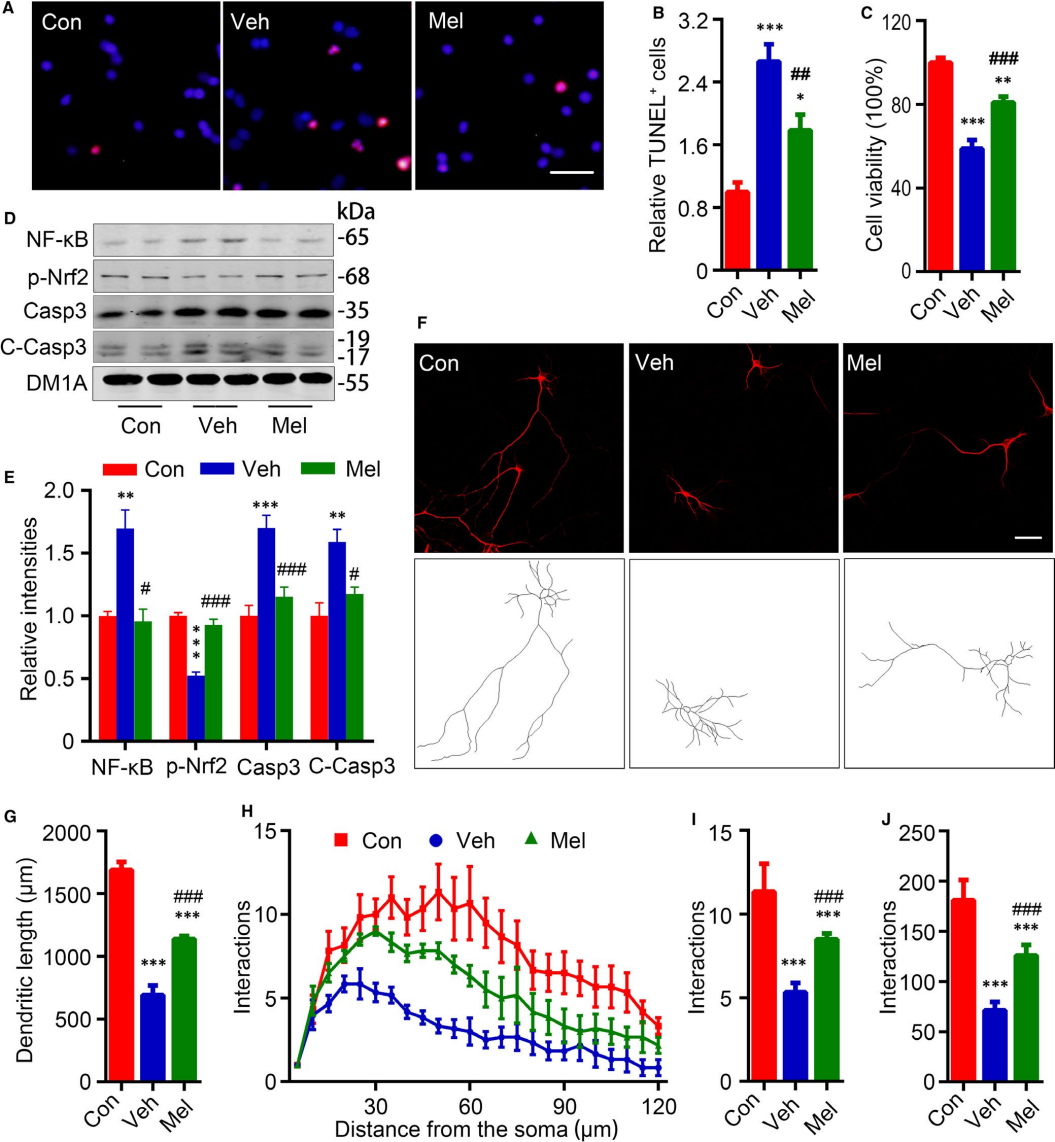
Melatonin impeded neuronal apoptosis and dendritic abnormalities in acidic condition. A, Neurons were treated with melatonin (Mel) (1 × 10^−4^ mol/L, Mel) or DMSO (0.01%, Veh) for 24 h, and TUNEL staining and CCK8 were performed to validate the cell death ratio and cell viability after pH6.2 for 24 h. TUNEL staining was performed after pH6.2 treatment for 24 h (Scale bar = 50 μm) and quantified (B). Data were presented as means ± SEM (n = 4/group). *P < 0.05, ***P < 0.001 vs Con, ^##^ *P *< 0.01 vs Veh. C, The relative cell viabilities of rat cortical neurons after pH6.2 for 24 h were shown. Data were presented as means ± SEM (n = 6/group). **P < 0.01, ***P < 0.001 vs Con, ^###^ *P *< 0.001 vs Veh. Levels of nuclear factor κ‐light‐chain‐enhancer of activated B cells (NF‐κB) p65, phosphorylated nuclear factor erythroid 2–related factor 2 (Nrf2) at Ser40 (p‐Nrf2), caspase‐3 (Casp3) and cleaved caspase‐3 (C‐Casp3) were measured by Western blotting (D) and quantitatively analysed (E). Data were presented as means ± SEM (n = 4/group). **P < 0.01, ***P < 0.001 vs Con, ^#^ *P *< 0.05, ^###^ *P *< 0.001 vs Veh. F, Representative images for observing neurons were showed after MAP2 staining (Scale bar = 50 μm). G, Total dendritic length was quantified using the software ImageJ loaded with the simple neurite tracer analysis plug‐in. H, Neuronal dendritic complexity was analysed using ImageJ software and Sholl analysis plug‐in, and maximum number of dendritic intersections (I) and sum of total dendritic intersections (J) were quantified. Data were presented as means ± SEM (n = 6/group). ***P < 0.001 vs Con, ^###^ *P *< 0.001 vs Veh

After growing in the normal medium for 13 days, the primary neurons were exposed in melatonin (1 × 10^−4^ mol/L, Mel) or DMSO (0.01%, Veh) for 24 hours. Then, Mel neurons and Veh neurons were cultured in the medium of pH6.2 for the next 24 hours. The synchronously cultured primary neurons in the normal medium were used as control (Con). The Mel neurons (1 × 10^−4^ mol/L) had much less apoptotic neurons (Figure 3A,B) and higher cell viabilities than Veh neurons in the medium of pH6.2 (Figure 3C). We also tested the levels of caspase‐3 (Casp3) and cleaved caspase‐3 (C‐Casp3) by Western blotting, and the levels of caspase‐3 and cleaved caspase‐3 in the Veh neurons were significantly higher than Con neurons and Mel neurons (Figure 3D,E). NF‐κB is a major effector mediating apoptosis in response to oxidative stress,^31^ and Nrf2 is a key factor in combating oxidative stress.^32^ In this research, an increasing of 69.6% in NF‐κB p65 and a decreasing of 47.5% in phosphorylated Nrf2 (p‐Nrf2, activation form) were detected by Western blotting in Veh neurons when compared to Con neurons. In Mel neurons, the levels of NF‐κB p65 and p‐Nrf2 returned to the levels as Con neurons had (Figure 3D,E).

By immunofluorescence staining of MAP2, the morphological changes of the neurons were analysed. Con neurons had a total dendritic length of 1690.0 ± 63.64 μm, while the Veh neurons had a significantly decreased total dendritic length (691.6 ± 77.25 μm) (Figure 3F,G). By Sholl analysis, we studied the dendritic complexities of neurons. In the concentric circle analysis, the Veh neurons had obviously decreased maximum number of dendritic intersections (5.3 ± 0.56, Figure 3H,I) and fewer total dendritic interactions (71.3 ± 8.31, Figure 3H,J) compared to Con neurons (181.0 ± 20.01 and 11.3 ± 1.67, respectively). Mel neurons had a longer total dendritic length and more dendritic intersections than Veh neurons (1139.0 ± 23.15 μm) (Figure 3H‐J).

### 3.3 Melatonin attenuates acidosis‐induced synaptic abnormality and tau hyperphosphorylation

**FIGURE 4:**
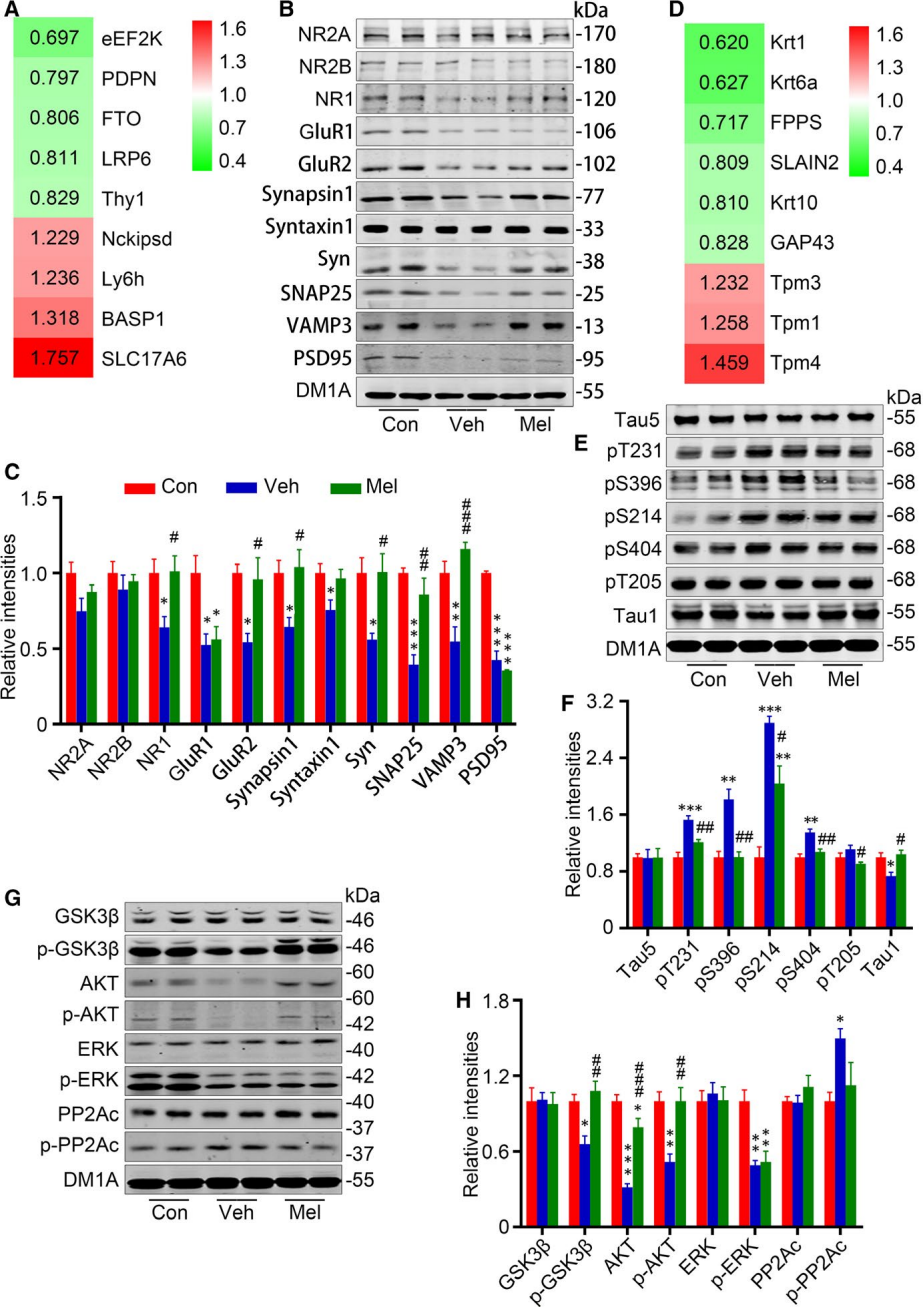
Melatonin attenuates acidosis‐induced synaptic abnormality and tau hyperphosphorylation. A, The differentially expressed proteins related to synaptic‐associated proteins are listed with a ratio (pH6.2/Con), including solute carrier family 17 (sodium‐dependent inorganic phosphate cotransporter), member 6 (SLC17A6), brain acid–soluble protein 1 (BASP1), lymphocyte antigen 6 family member H (Ly6h), thy‐1 membrane glycoprotein (Thy1), eukaryotic elongation factor‐2 kinase (eEF2K), LDL receptor–related protein 6 (LRP6), α‐ketoglutarate‐dependent dioxygenase FTO (FTO), podoplanin (PDPN) and Nckipsd. Red colour indicates the increased proteins (*P *< 0.05), and green indicates the decreased ones (*P *< 0.05). To investigate the alterations of synapse‐related proteins, levels of N‐methyl‐D‐aspartate receptor (NR) 2A (NR2A), NR2B, NR1, glutamate receptor (GluR) 1 (GluR1), GluR2, synapsin1, syntaxin1, synaptophysin (Syn), synaptosomal‐associated protein 25 (SNAP25), vesicle‐associated membrane protein 3 (VAMP3) and post‐synaptic density protein 95 (PSD95) were measured by Western blotting (B) and quantitatively analysed (C). D, The differentially expressed proteins related to cytoskeletal system are listed with a ratio (pH6.2/Con), including keratins (Krt1, Krt6a and Krt10), tropomyosins (Tpm1, Tpm3 and Tpm4), neuromodulin (GAP43), SLAIN2 and farnesyl pyrophosphate synthase (FPPS). Red colour indicates the increased proteins (*P *< 0.05), and green indicates the decreased ones (*P *< 0.05). Levels of total tau (Tau5), dephosphorylated tau (Tau1) and phosphorylation at Thr231 (pT231), Ser396 (pS396), Ser214 (pS214), Ser404 (pS404) and Thr205 (pT205) of tau were measured by Western blotting (E) and quantitatively analysed (F). To detect the alterations of tau hyperphosphorylation‐related kinases as well as their activity‐related phosphorylation, levels of glycogen synthase kinase 3β (GSK3β), phosphorylated GSK3β at Ser9 (p‐GSK3β), protein kinase B (AKT), phosphorylated AKT at Ser473 (p‐AKT), extracellular‐signal‐regulated kinases (ERK), and phosphorylated ERK at Thr202/Thr204 (p‐ERK), protein phosphatase 2A (PP2A) catalytic subunit (PP2Ac) and its Tyr307‐phosphorylation (p‐PP2Ac) were measured by Western blotting (G) and quantitatively analysed (H). Data were presented as means ± SEM (n = 4/group). *P < 0.05, **P < 0.01, ***P < 0.001 vs vs Con, ^#^ *P *< 0.05, ^##^ *P *< 0.01, ^###^ *P *< 0.001 vs Veh

In the differentially expressed proteins, several synaptic‐associated proteins were included, for example solute carrier family 17 (sodium‐dependent inorganic phosphate cotransporter), member 6 (SLC17A6), brain acid–soluble protein 1 (BASP1), lymphocyte antigen 6 family member H (Ly6h), thy‐1 membrane glycoprotein (Thy1), eukaryotic elongation factor‐2 kinase (eEF2K), LDL receptor–related protein 6 (LRP6), α‐ketoglutarate‐dependent dioxygenase FTO (FTO), podoplanin (PDPN) and Nckipsd (Figure 4A). SLC17A6, which encodes vesicular glutamate transporter 2 (vGluT2), mediates the uptake of glutamate into synaptic vesicles at presynaptic nerve terminals of excitatory neural cells.^33^ BASP1 is a presynaptic membrane protein interacting with other proteins as well as with lipids and contributing to in axon guidance, neuroregeneration and synaptic plasticity.^34^ Ly6h, which was found in most hippocampal pyramidal neurons, is involved in the regulation of α7 nicotinic acetylcholine receptors transport and nicotine‐induced glutamate signalling enhancement.^35^ eEF2K, also known as calcium/calmodulin‐dependent protein kinase III (CaMKIII), is involved in changes in synaptic plasticity and learning and memory.^36^, ^37^ Knockdown of eEF2K by RNA interference reduced the dendritic spine stability and inhibited the expression of dendritic brain‐derived neurotrophic factor (BDNF).^38^ LRP6 is a coreceptor for Wnt signalling, and its deficiency was reported contributes to synaptic abnormalities.^39^ FTO, a member of the Fe (II)‐ and 2‐oxoglutarate‐dependent AlkB dioxygenase family, plays important roles in neurogenesis as well as in learning and memory.^40^ PDPN, a cell‐surface glycoprotein constitutively expressed in the brain, is required for proper hippocampus‐dependent learning and memory functions. Deletion of PDPN selectively impairs activity‐dependent synaptic strengthening at the neurogenic dentate gyrus and hampers neuritogenesis.^41^ Nckipsd, also named SH3 protein interacting with Nck, 90kDa (SPIN90), is an Nck‐interacting protein highly expressed in synapses, is essential for actin remodelling and dendritic spine morphology. And SPIN90‐knockout mice exhibited substantial deficits in synaptic plasticity and behavioural flexibility.^42^ The pH6.2 neurons had the increased levels of SLC17A6 (1.757, *P* = 0.0088), BASP1 (1.318, *P* = 0.0039), Ly6h (1.236, *P* = 0.00013) and Nckipsd (1.229, *P* = 0.032), and the decreased Thy1 (0.829, *P* = 0.0020), eEF2K (0.697, *P* = 0.016), LRP6 (0.811, *P* = 0.028), FTO (0.806, *P* = 0.050) and PDPN (0.797, *P* = 0.012), indicating the synaptic abnormalities (Figure 4A).

Here, we made further detections of synaptic proteins (synapsin1 and synaptophysin (Syn), post‐synaptic density protein 95 (PSD95), glutamate receptor (GluR) 1 (GluR1), GluR2, N‐methyl‐D‐aspartate receptor (NR) 1 (NR1), NR2A and NR2B) and SNARE complex proteins (syntaxin1, synaptosomal‐associated protein 25 (SNAP25) and vesicle‐associated membrane protein 3 (VAMP3)). Compared with Con neurons, Veh neurons had significantly decreased levels of Syn (55.9%), synapsin 1 (64.4%), PSD95 (42.3%), GluR1 (52.5%), GluR2 (54.1%), NR1 (64.1%), syntaxin1 (75.5%), SNAP25 (39.5%) and VAMP3 (54.6%), and unchanged levels of NR2A and NR2B (Figure 4A,B). Mel neurons had higher levels of NR1 (157.6%), GluR2 (177.0%), synapsin1 (161.3%), Syn (158.9%), SNAP25 (217.1%) and VAMP3 (212.2%) than Veh neurons, while the levels of GluR1 and PSD95 remained low (Figure 4B,C). These data confirmed the synaptic impairments of neuron in the medium of pH6.2 and the protection of melatonin.

The stability of the cytoskeletal system is important for the function and morphology of neurons. Cytoskeletal proteins including keratins (Krt1, Krt6a and Krt10), tropomyosins (Tpm1, Tpm3 and Tpm4), neuromodulin (GAP43), SLAIN2 and farnesyl pyrophosphate synthase (FPPS) were included in the differentially expressed proteins (Figure 4D). Tropomyosins are a large family of integral components of actin filaments that play a critical role in regulating the function of actin filaments.^43^ GAP43, a protein expressed at high levels in neuronal growth cones during development and axonal regeneration, is a crucial component of the axon and presynaptic terminal.^44^ SLAIN2, a microtubule plus‐end tracking protein, has been identified as the regulator of microtubule dynamics and controls the axonal development and microtubule growth.^45^, ^46^ FPPS is the gate‐keeper of mammalian isoprenoids, which was indicated in tau phosphorylation.^47^ The pH6.2 neurons had increased levels of Tpm1 (1.258, *P* = 0.005), Tpm3 (1.232, *P* = 0.00001) and Tpm4 (1.459, *P* = 0.038), and decreased levels of Krt1 (0.62, *P* < 0.00001), Krt6a (0.627, *P* = 0.014), Krt10 (0.81, *P* = 0.00079), GAP43 (0.828, *P* < 0.00001), SLAIN2 (0.087, *P* = 0.040) and FPPS (0.717, *P* = 0.022), indicating the cytoskeletal abnormalities (Figure 4D).

It is well established that hyperphosphorylated tau, a MAP, induces morphological and functional abnormalities of neurons.^48^, ^49^ By Western blotting, we found the Veh neurons had higher levels of phosphorylated tau at Thr231 (pT231, 152.6%), Ser396 (pS396, 181.6%), Ser214 (pS214, 289.6%) and Ser404 (pS404, 134.9%), and a lower level of non‐phosphorylated tau at Ser198/199/202 (recognized by Tau1) than Con neurons, while Mel neurons only had the higher level of phosphorylated tau at Ser214 (Figure 4E,F). In addition, no evident changes were found in the levels of total tau probed by Tau5.

Lots of protein kinases (PKs) and phosphatases (PPs) are reported involved in the cytoskeletal abnormalities including hyperphosphorylation of tau.^50^ In the proteomic data, the protein kinases including mitogen‐activated protein kinase signal proteins (MAP4K4, MAP4K3, Map4k5, Map3k2, Map3k4, Map3k5, Map3k7, Map2k1, Map2k2, Map2k4, Map2k6, Mapk1, Mapk3, Mapk8, Mapk9, Mapk10 and Mapk14), cyclin‐dependent kinases (CDK4, CDK5, CDK6, CDK7, CDK9, CDK11, CDK16 and CDK17), glycogen synthase kinase‐3 (GSK3α and GSK3β), PKA, PKC (α, β and δ), phosphatidylinositol 3‐kinase (PI3Krβ and PI3Kcβ), calcium/calmodulin‐dependent protein kinase kinases (CAMKKI and CAMKKII) and calcium/calmodulin‐dependent protein kinases (CAMKI, CAMKII and CAMKIV), were not included in the differentially expressed proteins. And the phosphatases, including serine/threonine‐protein phosphatase 1 (PP1)‐γ catalytic subunit (Ppp1cc), Ppp3cb, Ppp3cc, Ppp4c, Ppp5c, PP1 regulatory subunit 10 (Ppp1r10), Ppp4r1, Ppp6r1, PP2A catalytic subunit β (Ppp2cβ), PP2A 55 kDa regulatory subunit B (Ppp2r2b), Ppp2r2d, PP2A 65 kDa regulatory subunit A β (Ppp2r1β) and PP2B catalytic subunit α (Ppp3cα), were not included in the differentially expressed proteins too.

Among various enzymes, GSK3β is the most implicated kinase in tau hyperphosphorylation and PP2A is the most active phosphatase in tau dephosphorylation, respectively.^50^ By Western blotting, Veh neurons had lower levels of phosphorylated GSK3β at Ser9 (p‐GSK3β, inhibition form), protein kinase B (AKT), phosphorylated AKT at Ser473 (active form) and phosphorylated extracellular‐signal‐regulated kinases (ERK) at Thr202/Thr204 (p‐ERK, active form) than Con neurons (Figure 4G,H). The activity of PP2A is up‐regulated by dephosphorylation of its catalytic subunit (PP2Ac) at Tyr307 (p‐PP2Ac).^50^ Here, the level of phosphorylated PP2Ac at Tyr307 was increased in Veh neurons, indicating the inhibition of PP2A. Except the decreased phosphorylated ERK, the levels of the enzymes in Mel neurons showed no differences from Con neurons. The total levels of GSK3β, ERK and PP2Ac showed no differences between groups (Figure 4G,H).

### 3.4 Melatonin regulates organelle stress responses induced by acidosis

**FIGURE 5:**
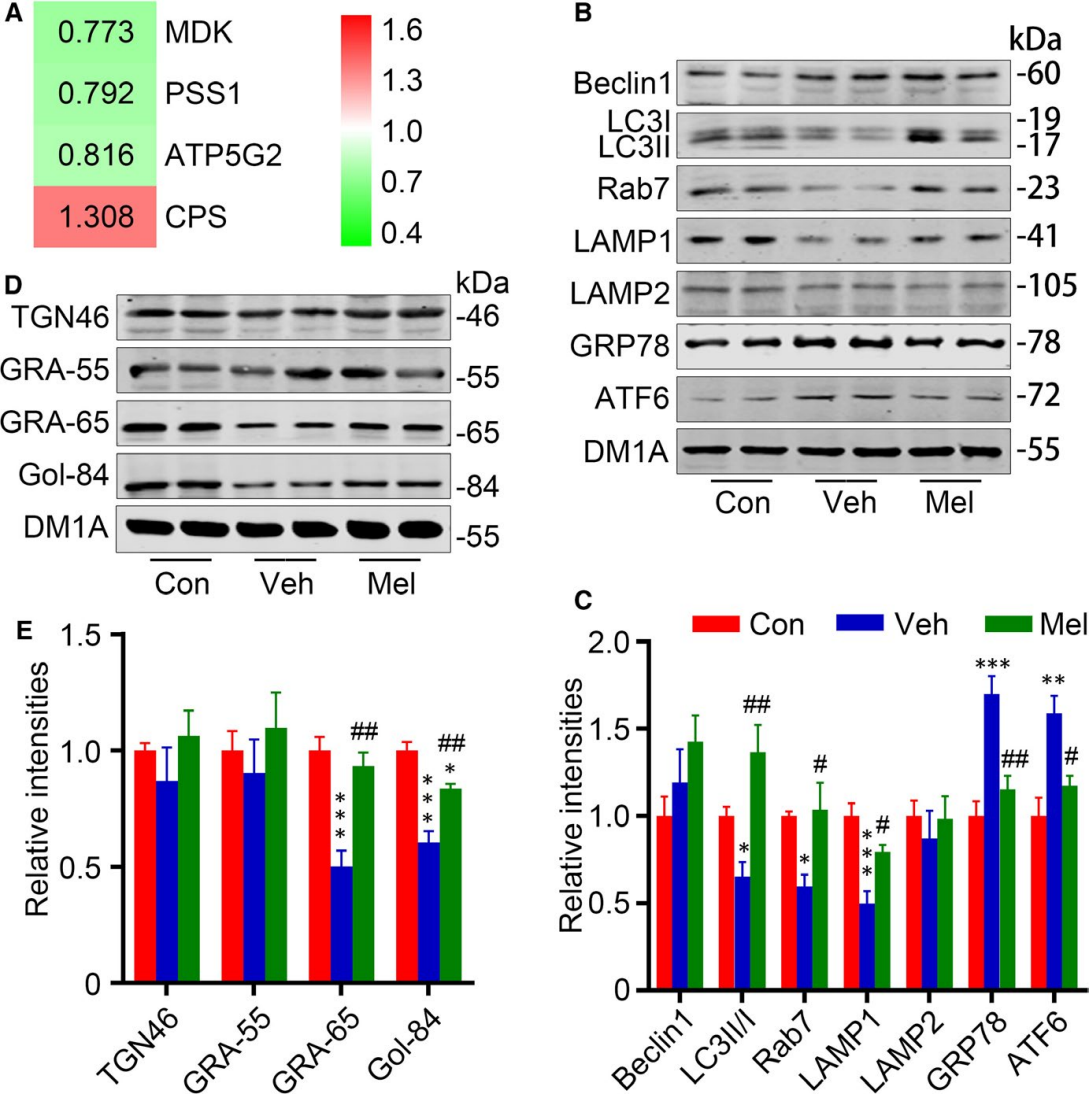
Melatonin regulates organelle stress responses induced by acidosis. A, The differentially expressed proteins related to organelle stress are listed with a ratio (pH6.2/Con), including ATP synthase F (0) complex subunit C2, mitochondrial (ATP5G2), carbamoyl phosphate synthetase 1 (CPS1), phosphatidylserine synthase 1 (PSS1) and midkine (MDK). Red colour indicates the increased proteins (*P *< 0.05), and green indicates the decreased ones (*P *< 0.05). Levels of Beclin1, microtubule‐associated protein light chain 3 (LC3), ras‐related protein rab‐7a (Rab7), lysosomal‐associated membrane protein 1 (LAMP1), LAMP2, glucose‐regulated protein 78 (GRP78/Bip) and activating transcription factor 6 (ATF6) were measured by Western blotting (B) and quantitatively analysed (C). Levels of Golgi matrix proteins including trans‐Golgi network protein 46 (TGN46), Golgi reassembly stacking protein 55 (GRASP55), GRASP65 and Golgi matrix proteins golgin‐84 (Gol‐84) were measured by Western blotting (D) and quantitatively analysed (E). Data were presented as means ± SEM (n = 4/group). *P < 0.05, **P < 0.01, *** *P *< 0.001 vs Con, ^#^ *P *< 0.05, ^##^ *P *< 0.01 vs Veh

Neuron has various organelles such as the endoplasmic reticulum (ER), Golgi apparatus (GA), mitochondria, lysosomes and peroxisomes. The organelle autoregulation, mainly including ER stress, Golgi stress, mitochondrial stress and lysosome stress, is indispensable for cells to regulate cellular functions. Mitochondria are double‐membrane‐bound subcellular organelles that provide a host of metabolic functions including energy production. Here, the ATP synthase F (0) complex subunit C2, mitochondrial (ATP5G2), was decreased in pH 6.2 neurons (0.816, *P* < 0.00001). Carbamoyl phosphate synthetase 1 (CPS1), the first rate‐limiting mitochondrial enzyme in the urea cycle, with increased levels indicating increased mitochondrial stress, was increased in pH 6.2 neurons (1.308, *P* = 0.00029). Phosphatidylserine synthase 1 (PSS1) is highly enriched in mitochondria‐associated membranes, which can facilitate phosphatidylserine (PS) import into mitochondria for decarboxylation to phosphatidylethanolamine, was decreased in pH 6.2 neurons (0.792, *P* = 0.021). Activation of AKT, Raf‐1 and PKC signalling, which supports neuronal survival and differentiation, requires interaction of these proteins with PS localized in the cytoplasmic leaflet of the plasma membrane.^51^ Midkine (MDK), a neurotrophic factor, which protects neurons from injury, was decreased in pH 6.2 neurons (0.773, *P* = 0.024). In neuronal cells, MDK activates the MAPK/ERK and PI3K/AKT pathways to promote cell survival and proliferation.^52^, ^53^ All these changes indicate the mitochondrial abnormality was induced by acidosis (Figure 5A).

Severe acidosis can be adversely affected not only by reducing antioxidant activity, but also by exerting pressure on the ER.^54^ A previous study indicated that ER stress plays an important role in activation of GSK3β.^55^ GRP78, a marker of ER stress, was increased in Veh neurons (170.0% of Con) (Figure 5B,C). Furthermore, ATF6, a transmembrane protein localized to the ER membrane and transported to the GA in a COP‐II vesicle upon ER stress, was increased in Veh neurons (158.9% of Con) (Figure 5B,C). The remarkable decreased levels of GRP78 and ATF6 were observed in Mel neurons compared to Veh neurons (67.9% and 74.0% of Veh, respectively) (Figure 5B,C).

Similar to the ER, the GA can sense and transduce death signals through its own unique molecular machinery in cell death pathways.^56^ It has also been reported that during apoptotic cell death, there is a crosstalk between ER, mitochondria and GA.^57^ GA is a central organelle of the secretory pathway, and its structure maintained by Golgi matrix proteins is important for the efficient processing of secretory cargo. As GA is disassembled after various cellular stresses, we detected the levels of several Golgi structural proteins, for example trans‐Golgi network protein 46 (TGN46), Golgi reassembly stacking protein 55 (GRASP55), GRASP65 and Golgi matrix proteins golgin‐84 (Gol‐84) by Western blotting. The levels of TGN46 and GRASP55 were not altered, while the levels of Gol‐84 and GRASP65 were significantly decreased in pH6.2 neurons (60.5% and 50.2% of Con, respectively), and these changes were restrained by melatonin (Figure 5D,E).

The lysosome is an organelle where proteins and polysaccharides are degraded and recycled by the mechanism called autophagy. Damaged biopolymers and organelles are engulfed by autophagosomes, which then fused with the lysosome. We observed the decreased levels of Rab7 and lysosomal‐associated membrane protein 1 (LAMP1) (59.7% and 50.0% of Con, respectively) (Figure 5B,C) in Veh neurons, which were significantly increased in Mel neurons (173.7% and 159.2% of Veh, respectively) (Figure 5B,C). Additionally, lysosome also acts as a signalling organelle that senses nutrient availability and generates an adaptive response, which is important for cellular homeostasis. It was reported lysosomal calcium release activates the master autophagy regulator transcription factor EB (TFEB).^58^ Here, levels of autophagy activation marker including beclin1 (autophagosome nucleation) and LC3II/I (autophagosome formation) showed no difference after acidic treatment. Macroautophagy (autophagy) is a highly conserved intracellular degradation system that is essential for homeostasis in eukaryotic cells. ER stress–triggered responses typically proceed in an autophagy‐dependent manner. Several autophagy markers including autophagy‐related 5 (ATG5, autophagosome elongation), beclin1, ATG2, ATG3, ATG4, ATG7, ATG9, ATG101, ATG13 and ATG16L showed no differences in the proteomic data of pH6.2 neurons. Furthermore, we assayed the ratio of LC3II/I, a marker of autophagosome formation, which was significant decreased in pH6.2 neurons and significantly increased in Mel neurons (209.3% of Veh) (Figure 5B,C). All these data suggested the autophagy‐lysosomal abnormality in pH6.2 neurons, and melatonin significantly reversed this abnormality.

## 4 DISCUSSION

In this research, we treated the cultured primary neurons with an acidic environment (pH6.2, 24 hours) to mimic the acidosis. By proteomic analysis, 69 differentially expressed proteins induced by acidosis were found, which were mainly related to stress and cell death, synaptic plasticity and gene transcription. The acidotic neurons developed obvious alterations including increased neuronal death, reduced dendritic length and complexity, reduced synaptic proteins, cytoskeletal abnormalities, ER stress activation, decreased Golgi matrix proteins, abnormal lysosomal signals and imbalanced oxidative stress/anti‐oxidative stress. Melatonin effectively reversed the acidosis‐induced neuronal injuries.

Acidosis is commonly associated with increased levels of inflammation, oxidative stress^59^, ^60^ and activating NF‐κB signalling.^61^ In cerebral ischaemia, NF‐κB signalling pathway plays an exceptionally important role due to its pleiotropic effects, unique regulatory mechanisms, and a large number of activating signalling pathways and genes it controls.^62^ Activating NF‐κB was shown to induce some cytotoxic factors to exacerbate inflammation and oxidative stress and promote apoptosis.^63^ Some organelles of neuron, such as the ER, mitochondria and lysosome,^64^, ^65^, ^66^ are susceptible to the reduction of pH and involved in the inflammation and oxidative stress–induced neuronal damage. Here, in the acidotic neurons with higher NF‐κB and lower phosphorylated Nrf2, we detected the ER stress activation, abnormal lysosome‐related signals, imbalanced oxidation/anti‐oxidation and decreased Golgi matrix proteins.

Dendrites are closely related to the morphology and number of synapses, and affect the induction of synaptic plasticity.^67^ In this study, the reduced length and complexity of dendrites and reduced synaptic proteins were shown in the acidotic neurons. The levels of Syn (a presynaptic marker), synapsin1 (a presynaptic marker) and PSD95 (a post‐synaptic marker) were significantly decreased, indicating the synaptic impairments of the neurons in the medium of pH6.2. In addition, significant decreases in SNARE complex proteins (including syntaxin1, SNAP25 and VAMP3) were also observed, suggesting the impaired docking and/or fusion of synaptic vesicles with the presynaptic membrane. It has been reported that acidosis impaired the spiking capacity in the GABAergic neurons more than in the glutamatergic neurons.^68^ Glutamate is the major excitatory neurotransmitter in the brain, and its receptors are divided into ionotropic glutamate receptors (iGluRs) and mGluRs. NMDARs comprise by NR1, NR2A, NR2B, NR2C and NR2D, and α‐amino‐3‐hydroxy‐5‐methyl‐4‐isoxazole propionic acid receptors (AMPARs) comprise by GluR1, 2, 3 and 4 are two important subtypes of the iGluRs family. Here, NR1, GluR1 and GluR2 were significantly decreased, and SLC17A6, which encodes vGluT2, was increased in the acidotic neurons. Our proteomic data also showed that several proteins related to glutamatergic synapse including glutamate decarboxylases (GAD1 and GAD2), GluR4, iGluR δ‐1 (GLUD1), GLUD2, glutamate receptor ionotropic kainate 2 (GRIK2), GRIK 3, GRIK5, glutamate receptor–interacting proteins (GRIP1 and GRIP2), mGluRs (mGluR1, mGluR5, mGluR7) and vGluT1 (SLC17A7) remained unchanged. Besides, several proteins related to GABAergic synaptic including GABAA receptor subunit β 1 (GABRB1), sodium‐ and chloride‐dependent GABA transporter 1 (SLC6A1), SLC6A3 and GABAB receptor 1 (GABBR1) also remained unchanged. Tau, a MAP, is important in stabilizing microtubules, and its hyperphosphorylation leads to the destabilization and dysfunctions of the microtubule network, and dysregulation in synaptic plasticity.^49^ Here, we found the neurons cultured in pH6.2 medium had much higher phosphorylation levels of tau at Thr231 (pT231), Ser396 (pS396), Ser214 (pS214) and Ser404 (pS404) sites. Abnormal hyperphosphorylation of tau is the result of imbalance of tau kinases and phosphatases. GSK3β is one major kinase, and PP2A is thought to be the major phosphatase for tau dephosphorylation in AD.^50^ In this research, inhibition of PP2A, AKT and ERK1/2 and activation of GSK3β were observed in acidotic neurons.

GSK3β was shown activated with the elevation of GRP78 during ER stress–induced tau hyperphosphorylation with spatial memory deficits in rats.^55^, ^69^ In this study, GRP78 and ATF6 were increased in the acidotic neurons, indicting the activation of ER stress. Furthermore, we observed decreased GRASP65, Gol‐84, LAMP1, Rab7 and LC3II/I in the acidotic neurons. Double knockout of GRASP65 and GRASP55 dispersed the Golgi stack into single cisternae and tubulovesicular structures.^70^ Pharmacological intervention or overexpression of the C‐terminal fragment of GRASP65 inhibited fragmentation and decreased or delayed neuronal cell death.^57^ In addition, our previous study has shown that down‐regulation of Gol‐84 induces Golgi fragmentation with hyperphosphorylation of tau.^71^ Decreased Sesn3, LC3II/I, LAMP1 and Rab7 indicated the dysregulation of autophagy‐lysosome signals. NF‐κB has an essential role in inflammation and is an important player in the pathophysiology of neuronal impairments, with roles in cell death and synaptic dysfunction.^72^, ^73^ It was found that there is a crosstalking between the NF‐κB pathway and GSK‐3β signalling pathways. GSK‐3β, which was initially identified as a key regulator of insulin‐dependent glycogen synthesis, is known to be a mediator of a number of major signalling pathways including the PI3K pathway, the Wnt pathway and Notch pathway. It was shown that GSK‐3β activation is required for NF‐κB activation,^74^ and GSK‐3β inhibition caused a dramatic decrease in NF‐κB activity.^75^ However, NF‐κB activation by tumour necrosis factor requires AKT activation.^76^ The exact molecular mechanism of GSK‐3β mediated NF‐κB modulation is still elusive and requires further clarification.

In summary, acidosis‐induced rat primary neurons underwent significant apoptosis, synaptic abnormality and tau hyperphosphorylation. Melatonin partially reversed acidosis‐induced neuronal death, abnormal dendritic complexity, reduced synaptic proteins, tau hyperphosphorylation and imbalance of kinase/phosphatase probably via rescuing GSK‐3β and NF‐κB activation. Additionally, acidosis‐induced ER stress, Golgi stress and abnormal autophagy‐lysosome signals were completely reversed by melatonin. In conclusion, we verified that melatonin has a protective effect on acidosis‐induced neuronal dysfunctions and ultimately decreases neuronal death from acidosis.

## CONFLICT OF INTEREST

The authors declare no conflict of interest.

## AUTHOR CONTRIBUTIONS

QT and X‐WZ initiated, designed and supervised the study. YS and E‐LC conducted most of the experiments, and CY and C‐YY performed the primary neuron culture. PZ and X‐MW did the statistical analysis. Y‐YF, Z‐KC, QW and F‐YC performed part of the molecular biological experiments. YS and E‐LC wrote the manuscript, and QT revised the manuscript.

## Data Availability

Data available on request from the authors.
