# Corrigendum

**DOI:** 10.1111/jcmm.15632

**Published:** 2020-09-28

In Shi et al,^1^ the published article contains errors in Figure 1. The correct figures are shown below. The authors confirm all results, conclusions of this article remain unchanged.

**FIGURE 1:**
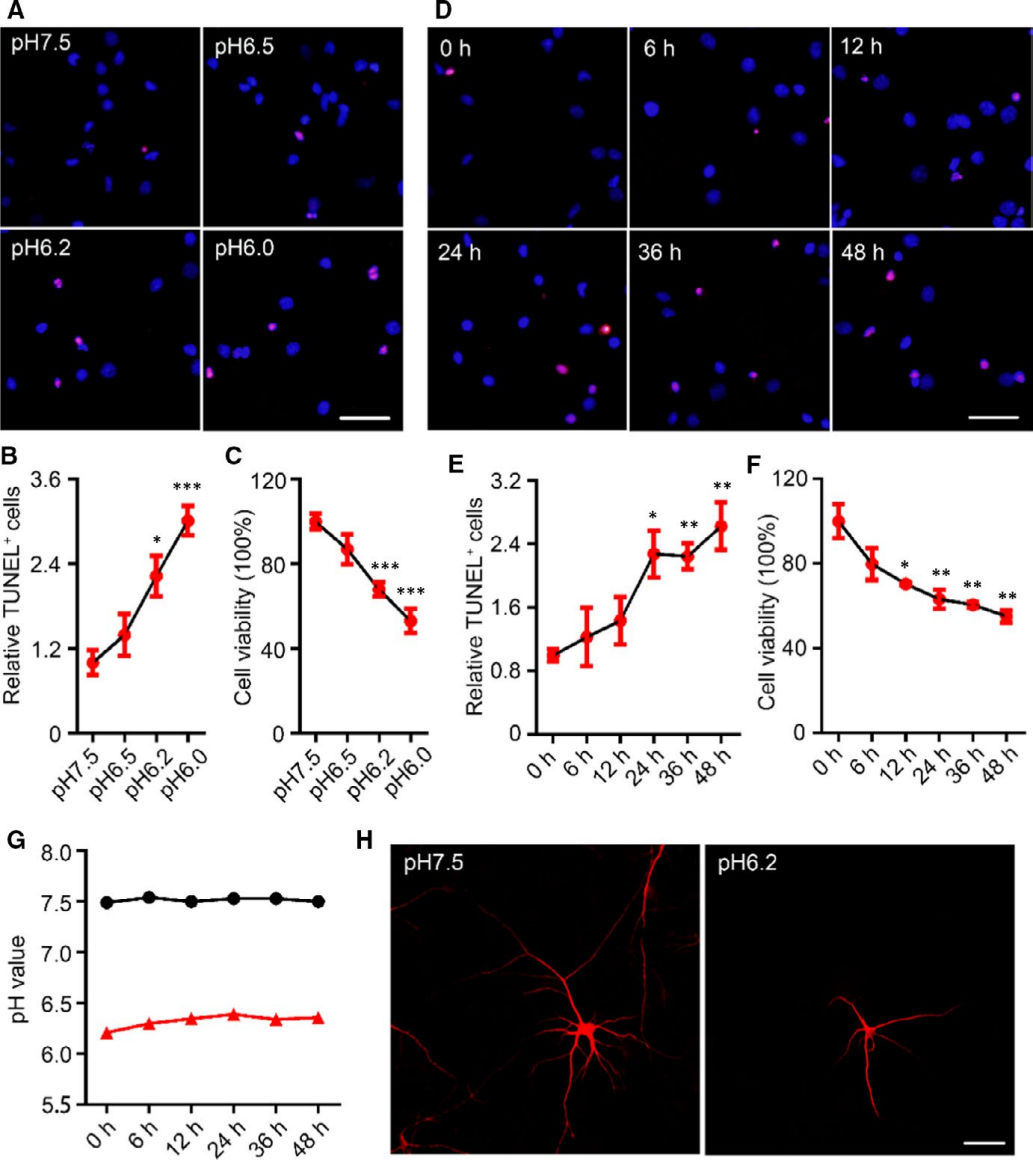
Decreases in extracellular pH value induced neuronal injury. A, TdT‐mediated dUTP nick‐end labelling (TUNEL) staining (red) in cultured rat cortical neurons (14‐d in vitro, 14 DIV) was performed after acid treatments (pH6.5, pH6.2 and pH6.0) for 24 h (scale bar = 50 μm) and quantified (B). Data were presented as means ± SEM. *P < .05, ***P < .001 vs Con, n = 4/group. C, The relative cell viabilities of rat cortical neurons after acidic treatment for 24 h were shown by Cell Counting Kit‐8 (CCK8) assay. Data were presented as means ± SEM. ***P < .001 vs Con, n = 6/group. D, TUNEL staining in the rat cortical neurons (14 DIV) was performed after pH6.2 treatment for 0‐48 h (eg 0, 6, 12, 24, 36 and 48 h, respectively) (scale bar = 50 μm) and quantified (E). Data were presented as means ± SEM. *P < .05, **P < .01 vs Con, n = 4/group. F, The relative cell viabilities of rat cortical neurons after pH6.2 treatment for 0‐48 h were shown by CCK8 assay. Data were presented as means ± SEM. *P < .05, **P < .01 vs Con, n = 6/group. G, Changes in acidity of normal medium and pH6.2 medium within 48 h. H, Images for observing neurons were collected on a confocal laser scanning microscope after immunofluorescence staining with an antibody recognizing microtubule‐associated protein 2. Scale bar = 50 μm
